# Prognostic Significance of Incidental Deep Vein Thrombosis in Patients with Cancer Presenting with Incidental Pulmonary Embolism

**DOI:** 10.3390/cancers12082267

**Published:** 2020-08-13

**Authors:** Maria Barca-Hernando, Rocio Ortega-Rivera, Sergio Lopez-Ruz, Teresa Elias-Hernandez, Maria Isabel Asensio-Cruz, Samira Marin-Romero, Javier Toral, Emilio Montero, Veronica Sanchez, Elena Arellano, Jose Maria Sanchez-Diaz, Macarena Real-Dominguez, Remedios Otero-Candelera, Luis Jara-Palomares

**Affiliations:** 1 Respiratory Department, Medical Surgical Unit of Respiratory Diseases, Hospital Virgen del Rocio, CIBERES, 41013 Sevilla, Spain; maria.barca.sspa@juntadeandalucia.es (M.B.-H.); rocio.ortega.rivera.sspa@juntadeandalucia.es (R.O.-R.); sergio.lopez.ruz.sspa@juntadeandalucia.es (S.L.-R.); teresa.elias.sspa@juntadeandalucia.es (T.E.-H.); isabel.asensio.cruz.sspa@juntadeandalucia.es (M.I.A.-C.); samira.marin.sspa@juntadeandalucia.es (S.M.-R.); rotero@separ.es (R.O.-C.); 2 Centro de Investigación Biomédica en Red de Enfermedades Respiratorias (CIBERES), Instituto de Salud Carlos III, 28029 Madrid, Spain; 3 Emergency Unit, Hospital Virgen del Rocio, 41013 Sevilla, Spain; jignacio.toral.sspa@juntadeandalucia.es (J.T.); emilio.montero.sspa@juntadeandalucia.es (E.M.); 4 Institute of Biomedicine of Seville (IBIS), Hospital Virgen del Rocio/CSIC/Universidad de Sevilla, 410013 Sevilla, Spain; veronica.sanchez.lopez.sspa@juntadeandalucia.es (V.S.); elena.arellano.orden.sspa@juntadeandalucia.es (E.A.); 5 Pharmacy, Respiratory Department, Medical Surgical Unit of Respiratory Diseases; Hospital Virgen del Rocio, 410013 Sevilla, Spain; josem.sanchez.diaz.sspa@juntadeandalucia.es; 6 Department of Preventive Medicine and Public Health, Universidad de Málaga, 29010 Malaga, Spain; macarenareal@uma.es

**Keywords:** neoplasm, incidental, pulmonary embolism, venous thromboembolism, mortality, prognosis

## Abstract

In symptomatic acute pulmonary embolism (PE), the presence of deep vein thrombosis (DVT) is a risk factor for 30- and 90-day mortality. In patients with cancer and incidental PE, the prognostic effect of concomitant incidental DVT is unknown. In this retrospective study, we examined the effect of incidental DVT on all-cause mortality in such patients. Adjusted Cox multivariate regression analysis was used for relevant covariates. From January 2010 to March 2018, we included 200 patients (mean age, 65.3 ± 12.4 years) who were followed up for 12.5 months (interquartile range 7.4–19.4 months). Of these patients, 62% had metastases, 31% had concomitant incidental DVT, and 40.1% (*n* = 81) died during follow-up. All-cause mortality did not increase in patients with DVT (hazard ratio [HR] 1.01, 95% confidence interval [CI] 0.43–2.75, *p* = 0.855). On multivariate analysis, weight (adjusted HR 0.96, 95% CI 0.92–0.99, *p* = 0.032), and metastasis (adjusted HR 10.26, 95% CI 2.35–44.9, *p* = 0.002) were predictors of all-cause mortality. In conclusion, low weight and presence of metastases were associated with all-cause mortality, while presence of concomitant DVT was unrelated to poorer survival.

## 1. Introduction

Venous thromboembolic (VTE) disease includes pulmonary embolism (PE) and deep venous thrombosis (DVT); however, these manifestations of the same disease have different clinical courses and prognoses [1,2]. The mortality rates for acute PE during the first three months of treatment have been reported to range from 1.4 to 17.4% [3,4]. Estimates derived from epidemiological data show the rate of incidental PE in the course of symptomatic DVT to be between 35% and 66% [5,6]. Using venography, Girard et al. found that 81.7% of patients with acute PE were found to have lower-extremity DVT [7]. Jiménez et al. reported that patients with concomitant DVT had, compared with those without DVT, an increased risk of all-cause death, supporting the hypothesis that an increased thrombus burden can lead to a poor prognosis [8]. However, several studies that have investigated the prognosis of concomitant DVT in patients with acute PE have reported conflicting data [3,8,9,10,11,12,13,14,15]. A meta-analysis showed that the presence of DVT was associated with a higher risk of death in the short-term for patients with symptomatic acute PE [16].

VTE is the main manifestation of the hypercoagulability state associated with cancer and is a leading cause of mortality and morbidity in patients with malignancies [17,18,19]. Several studies have reported the rate of an incidental diagnosis of PE in patients with cancer, with an incidence rate of unsuspected PE of 4 to 9% [20,21,22]. A meta-analysis of five studies involving patients with cancer calculated a weighted mean prevalence of unsuspected PE of 3.1% [23]. Nevertheless, few studies have focused on the prognostic effect of incidental concomitant DVT in patients with cancer and incidental PE [24,25,26]. In 2019, an observational, international, and prospective study assessed current treatment strategies for incidental PE in 695 patients with cancer [24]. This study did not provide data on the number of patients with incidental PE with associated DVT. In 2020, Mulder et al. published a post-hoc analysis of the Hokusai VTE-Cancer Clinical Trial [25], which analyzed 1046 patients with cancer and VTE, of whom 32% had incidental VTE [26]. Of them, 87.6% (*n* = 290) had PE with or without DVT. However, it was not possible to determine how many patients had concomitant incidental DVT.

We aimed to assess the incidence of concomitant incidental DVT in patients with cancer and incidental PE, and to evaluate the association between the presence of concomitant DVT and mortality risk in this group.

## 2. Results

**Figure 1:**
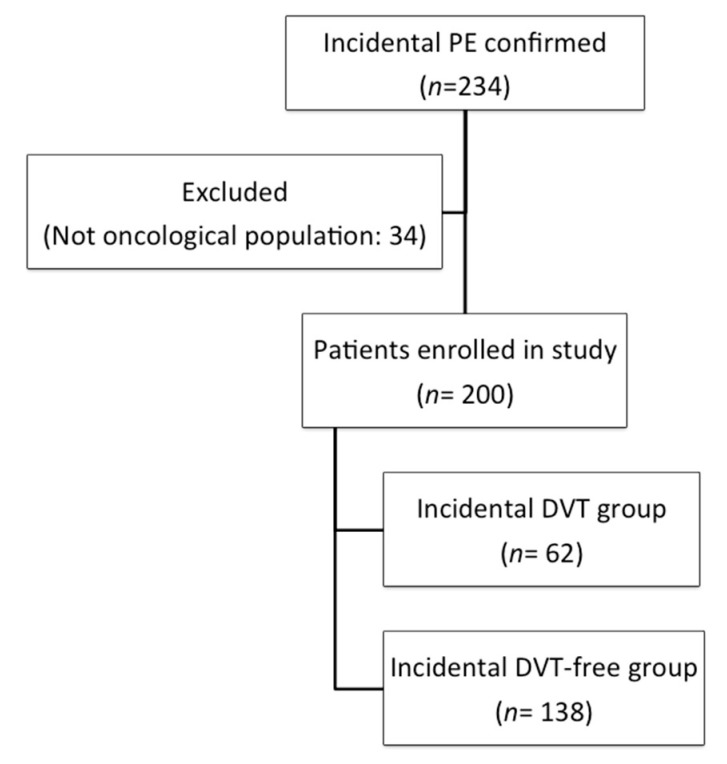
Flow diagram. Abbreviations: DVT: deep vein thrombosis; PE: pulmonary embolism.

In total, we evaluated 234 patients with incidental PE, 34 of whom were excluded because they did not have a diagnosis of cancer (Figure 1). In the final cohort comprising 200 patients, 62 (31%) patients had concomitant incidental DVT. The mean patient age was 65.3 ± 12.4 years (mean ± standard deviation), 62.2% had metastatic disease, and 67.5% were receiving oncological treatment at the time of the thrombotic event. All included patients had a histologically confirmed diagnosis of cancer, and the most observed cancer types were colorectal cancer (29.5%), lung cancer (17.5%), and breast cancer (8%). Baseline patient characteristics are shown in Table 1. The median duration from cancer diagnosis to VTE development was 5.8 months (interquartile range [IQR] 2.8–22.2 months). The median follow-up duration was 12.5 months (IQR 7.4–19.4 months). In total, 199 of 200 patients (99.5%) received low molecular weight heparin (LMWH) and most patients (198/199) received the therapeutic dose.

A comparison of patients with and without DVT indicated that those with concomitant DVT had a significantly higher prevalence of metastasis (72.4% vs. 57.5%, respectively; odds ratio [OR] 1.87, 95% confidence interval [CI] 1.01–3.19). Furthermore, at the time of the diagnosis of incidental PE, 75.8% of patients with concomitant DVT were receiving oncological treatment compared with 62.3% in the DVT-free group (OR 1.9, 95% CI 1.06–3.39). The most frequent cancer treatments involved antimetabolites (47.5%), followed by platinum-based agents (35%). Among patients with cancer who had undergone treatment, 22.5% had been prescribed one drug, 33% had been prescribed two drugs, and 11% had been prescribed ≥3 drugs. Patient characteristics according to survival (no-death vs. death) are shown in Table 2.

### 2.1. Primary and Secondary Outcomes

**Figure 2:**
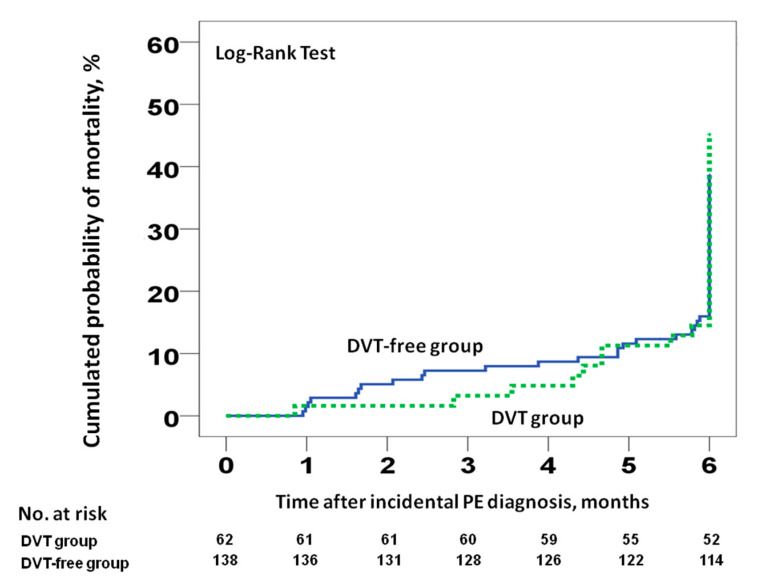
All-cause mortality at six months for patients with incidental PE, stratified according to the presence or absence of concomitant incidental DVT at the time of PE diagnosis. Abbreviations: DVT: deep vein thrombosis; PE: pulmonary embolism. Log-rank *p* > 0.05.

**Figure 3:**
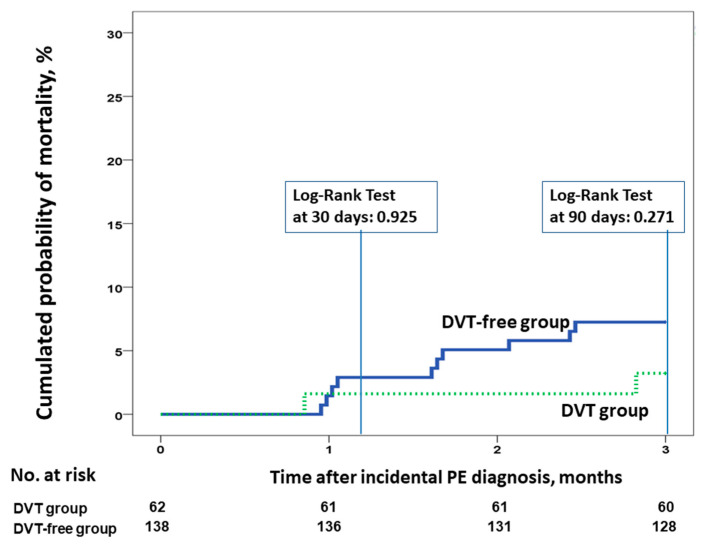
All-cause mortality at 30 and 90 days for patients with incidental PE, according to the presence or absence of concomitant incidental DVT at the time of PE diagnosis. Abbreviations: DVT: deep vein thrombosis; PE: pulmonary embolism. Log-rank *p* > 0.05.

Data were available for all patients at the end of the study. Of the 200 patients, 81 (40.5%) died during follow-up (95% CI 33.6–47.7%). Among them, 6% (95% CI 3.1–10.3%) and 15.5% (95% CI 10.8–21.3%) died at three and six months, respectively. Four patients (2%; 95% CI 0.5–5%) died of possible PE. There were 28 deaths (45.2%; 95% CI 32.5–58.3%) and 53 deaths (38.4%; 95% CI 30.3–47.1%) in patients with and without concomitant DVT, respectively. At six months, there were no differences in cumulative mortality in patients with incidental PE with and without incidental DVT (hazard ratio [HR] 1, 95% CI 0.42–2.41, *p* = 0.992; Figure 2). Similar results were found when we compared the 30- or 90-day all-cause mortality rates (Figure 3). There were no differences in the rate of VTE recurrence in patients with and without concomitant DVT (9.7% vs. 10.9%, HR 0.88, 95% CI 0.38–0.2, *p* = 0.97).

In the univariate analyses, weight (per kilogram) (HR 0.95, 95% CI 0.91–0.99, *p* = 0.024) and the presence of metastases (HR 9.12, 95% CI 1.96–42.34, *p* = 0.005) at the time of incidental PE diagnosis showed significant associations with death during follow-up (Table 3). In the multivariate analyses, weight (per kilogram) (adjusted HR 0.96, 95% CI 0.932–0.99, *p* = 0.032) and the presence of metastases (adjusted HR 10.26, 95% CI 2.35–44.9, *p* = 0.005) continued to show associations with poor prognoses.

### 2.2. Venous Thromboembolism (VTE) Recurrence and Bleeding

VTE recurrence was observed in 21 patients (10.5%, 95% CI 6.62–15.6%), and among them, 12 (57%) were symptomatic. Most (53%) cases of VTE recurrence were associated with PE. Bleeding occurred in 15 patients (7.5%, 95% confidence interval [CI] 4.3–12.1), and major bleeding occurred in two patients (1%, 95% CI 0.12–3.57%).

## 3. Discussion

In this study of patients with cancer and incidental PE, the presence of concomitant incidental DVT was not associated with poorer survival rates. One-third of patients with cancer and incidental PE also had incidental DVT. The prevalence of concomitant DVT in the study cohort (31%) was slightly lower than that reported in a series of patients with acute PE without cancer, in which the prevalence of ultrasound-detectable concomitant DVT varied from 39 to 63% [3,8,27]. This difference may be explained by the fact that the thrombotic load in patients with incidental PE is lower. Data on patient age and sex as well as on cancer types observed in our population were found to be similar to those reported in other studies [24]. The incidence of incidental PE in patients with cancer has been reported to range from 0.36 to 14.9%, depending on various factors such as cancer type, number of detector rows in the CT scanner, and slice thickness [28]. 

Several recent studies have assessed the prognostic effect of concomitant DVT in patients with acute PE [8,9,16,29]. One study, involving 2442 patients registered in the ICOPER (International Cooperative Pulmonary Embolism Registry) who had acute PE, did not observe an association between the presence of DVT and all-cause mortality [3]. Girad et al. observed that concomitant DVT was not associated with the risk of death at three months [9]. However, a study involving 707 patients with acute PE and concomitant DVT reported higher cumulative mortality than that observed in patients without DVT [8]. In a smaller study of 296 patients with symptomatic acute PE, Wicki et al. showed that the presence of concomitant DVT was associated with a higher mortality risk [29]. Becattini et al. performed a meta-analysis of studies that enrolled patients with acute PE to assess the prognostic value of concomitant DVT in terms of primary (30-day all-cause mortality) and secondary outcomes (90-day PE-related adverse events) [16]. They found that of the 8859 patients enrolled from across nine studies, 56% had concomitant DVT. Concomitant DVT was significantly associated with 30-day all-cause mortality (OR 1.9, 95% CI 1.5–2.4), although no association was found with 90-day PE-related adverse outcomes. These studies of patients with symptomatic acute PE analyzed all-cause mortality at 30 or 90 days. Meanwhile, our study is the first to analyze the effect of incidental DVT in patients with incidental PE, and we proposed all-cause mortality at six months (as the primary outcome) and at 30 and 90 days. However, we found no association between the presence of DVT and all-cause mortality in any of the analyses performed. 

There is a paucity of data concerning patients with incidental PE compared with those with symptomatic acute PE. Although current evidence is largely derived from observational and retrospective data, incidental VTE appears to be associated with prognoses, in terms of recurrent VTE development, bleeding, and mortality, which are as poor as those for symptomatic VTE [28]. In 2019, Kraaijpoel et al. conducted an international prospective, observational cohort study involving 695 patients with active cancer and a recent diagnosis of incidental PE. The 12-month cumulative incidence rates of recurrent VTE, major bleeding, and death were 6% (95% CI 4.4–8.1%), 5.7% (95% CI 4.1–7.7%), and 43% (95% CI 39–46%), respectively [24]. Similar findings were also obtained in a post-hoc analysis conducted as part of the Hokusai VTE Cancer Study [25], and the 12-month cumulative incidence rates of recurrent VTE and major bleeding were reported to be 7.9% and 6.6%, respectively [26]. However, none of these studies provided detailed information on how many patients had incidental DVT nor did they analyze the effect of incidental DVT on the survival of patients with incidental PE.

In our study, metastases were associated with a higher frequency of incidental DVT (OR 1.87, 95% CI 1.01–3.19). This finding is in line with previous observations in patients with cancer and VTE. An analysis of the California Cancer Registry found that the incidence of VTE was higher among patients with metastatic disease [30]. This can be explained by the hypercoagulability state associated with the presence of metastases [31]. On analyzing the survival values of our patients, we found that those with incidental PE and metastases had a significantly lower short-term survival rate than those without metastases. This finding is consistent with the results reported by Den Exter et al., who used data from the Registro Informatizado de la Enfermedad TromboEmbólica and showed that the presence of metastases was significantly associated with 30-day mortality [32].

The associations between weight, body mass index (BMI), and survival outcomes have previously been shown in other studies [33,34,35,36]. A meta-analysis that examined almost 100 studies involving >2.88 million patients confirmed that being overweight (BMI 25–30 kg/m^2^) was associated with a lower risk of all-cause mortality [33]. In a retrospective study of 3799 patients diagnosed with colorectal cancer, Shahjehan et al. reported that patients who were underweight had an increased risk of mortality [34]. Kang et al. evaluated BMI as a prognostic factor for 276 patients with advanced biliary tract cancer treated with palliative chemotherapy, and concluded using their multivariate analysis that patients who were overweight had a reduced risk of mortality (HR 0.632, 95% CI 0.436–0.918, *p* = 0.016) [35]. The role of weight in patients with metastases has been evaluated in a retrospective study to investigate the potential association between BMI and survival in patients with distant metastases (*n* = 4010) [36]. That study found that, compared with patients with healthy weight, individuals who were obese (HR 0.676, *p* < 0.001) and overweight (HR 0.84, *p* < 0.001) had a lower risk of all-cause mortality. Similar results were found in patients with VTE in a registry study that included 1642 patients with VTE and morbid obesity and 18,484 patients with healthy weight [37]. The final multivariable analysis showed a lower risk of death in patients with morbid obesity with cancer (HR 0.68, 95% CI 0.5–0.94) and without cancer (HR 0.67, 95% CI 0.49–0.96). In our study, we observed a poorer survival in patients with lower weight. However, we had no data on patient height, so we could not calculate the BMI. Nevertheless, our findings regarding the presence of a correlation between low weight and low BMI are consistent with those of previous studies.

Our study had several strengths. First, once we had obtained initial evidence indicating that the presence of DVT could be associated with a poorer prognosis in patients with symptomatic acute PE, we then included performing a compression ultrasound of the lower limbs in patients with PE in the protocol. This allowed for the identification of incidental DVT in this population. Second, on analyzing the variables associated with mortality among patients with incidental PE, we included the most relevant factors, and we were then able to perform a multivariate-adjusted analysis.

Our study also had some limitations. First, its single-center design may have limited the external validity of the results. Second, we did not find an increase in the mortality rates among the patients with incidental DVT. Even so, cancer is associated with several other factors that have a more decisive influence on patient survival (e.g., metastases); therefore, for patients with cancer, there are many factors that could have had an effect on survival and that could mitigate the effect of the presence of DVT on survival. It is also possible that the sample size may not have been sufficiently large to identify differences in both groups (in terms of study power), and for that reason, larger prospective cohorts are needed. Third, the retrospective nature of the study could be associated with information bias; however, as above-mentioned, per protocol, we performed compressive ultrasound in patients with PE. Cancer types were not included in the model because the number of cancer locations was high and the number of patients in terms of the various locations was limited. We had no data on thrombophilic risk factors, although the association of thrombophilia with worse survival was not observed in the above-mentioned studies. We analyzed the role of weight as a predictor of death. A BMI analysis would also have been interesting; however, as we had no data on patient height, we were unable to analyze this variable. Although the incidence of thrombosis was low, we did not review the CT scans of the patients to determine whether thrombosis had occurred in other locations (e.g., splanchnic vein thrombosis, inferior vena cava thrombosis, gonadal vein thrombosis, superior vena cava thrombosis, brachiocephalic vein thrombosis). Instead, our study focused on the effect of incidental DVT present in the lower limbs.

## 4. Materials and Methods

### 4.1. Study Design

This retrospective study used prospective data from consecutive inpatients and outpatients with VTE. The study was conducted in accordance with the Declaration of Helsinki, and the study protocol was approved by the Ethics Committee of Virgen del Rocio Hospital, Sevilla, Spain (Approval No. 1283-N-18). The requirement for written patient informed consent was waived due to the study design.

### 4.2. Setting

The participants of this study were recruited from among the inpatients and outpatients of a respiratory ward at a teaching hospital in Sevilla, Spain, between January 2010 and March 2018.

### 4.3. Eligibility 

This study included consecutive patients with an objectively confirmed diagnosis of incidental PE, based on a positive chest computed tomography (CT) result. Patients who were unable to attend follow-up due to geographical challenges and those aged <18 years old were excluded. Histological confirmation was required for all patients diagnosed with cancer. Major bleeding was assessed according to the following International Society of Thrombosis and Hemostasis (ISTH) criteria: fatal bleeding, and/or bleeding in a critical area or organ such as intracranial, intraspinal, intraocular, retroperitoneal, intra-articular or pericardial, or intramuscular organs with compartment syndrome, and/or bleeding causing a fall in hemoglobin level of ≥20 g/L or leading to transfusion of two or more units of whole blood or red blood cells [38]. The Eastern Cooperative Oncology Group (ECOG) performance status was used as a scale to assess the patient’s disease progression, to assess how the disease affected the patient’s activities of daily living, and to determine appropriate treatment and prognosis.

### 4.4. Lower Limb Ultrasound

Once the patients were diagnosed with PE, trained staff performed bilateral proximal and distal lower limb deep venous system compression ultrasonography in all patients at the time of their hospital consultation. The diagnostic criterion for DVT was the non-compressibility of a region within the deep venous system. 

### 4.5. Study Endpoints and Outcome Measures

In this study, all-cause mortality at six months after the diagnosis of PE was the primary endpoint. The secondary endpoints were PE-related mortality, VTE recurrence, and all-cause mortality at 30 and 90 days. Two investigators (Ortega Rivera and Jara-Palomares) reviewed and assessed all the outcomes. 

### 4.6. Statistical Analysis

Quantitative variables were expressed as means ± standard deviations. Medians and interquartile ranges (IQR) were used for non-normally distributed continuous data, and numbers and proportions for categorical data. We calculated Kaplan–Meier probabilities to estimate time to death and time to VTE recurrence, and a log-rank test was performed to assess differences between the groups. We censored for loss to follow-up (none) and study completion in the survival analyses. In addition, we controlled VTE recurrence models censored for death. To evaluate the association between concomitant DVT at the time of PE presentation, we performed a Cox proportional hazard regression analysis. In the full model, we included variables with a potential prognostic role in addition to the presence of incidental DVT. Backward stepwise regression was carried out to obtain the adjusted final model, with the variables selected in the equation in the last step. For all analyses, statistical significance was defined at a *p*-value of <0.05. The 95% CI was calculated using the Clopper–Pearson exact method. Binomial distribution using Statistical Package for the Social Sciences (SPSS) Statistics version 20.0 (IBM, Chicago, IL, USA) was employed, and all statistical analyses were performed using IBM SPSS Statistics version 24.0 software.

## 5. Conclusions

This study revealed that in patients with cancer with incidental PE, the presence of concomitant incidental DVT was not associated with poorer survival outcomes. One-third of all cancer patients with incidental PE were more likely to have an incidental DVT. The variables associated with all-cause mortality were low weight and the presence of metastases.

## Figures and Tables

**Table 1:** A comparison of baseline characteristics in the deep vein thrombosis (DVT) and DVT-free groups.

Variable	DVT Group (*n* = 62)	DVT-Free Group (*n* = 138)	Patients (*n* = 200)
Male sex, *n* (%)	34 (54.8)	80 (58)	114 (57)
Age (years), mean (SD)	66.6 (10.9)	64.7 (12.9)	65.3 (12.4)
Weight (kg), mean (SD)	75.5 (11.2)	72.6 (5.1)	73.6 (14.1)
Metastases, *n* (%)	42 (72.4)	73 (57.5)	115 (62.2)
Oncological treatment at VTE event, *n* (%)	47 (75.8)	86 (62.3)	133 (66.5)
Alkylating agents	5 (8.1)	1 (0.7)	6 (3)
Platinum-based agents	21 (33.9)	49 (35.5)	70 (35)
Topoisomerase inhibitors	7 (11.3)	19 (13.8)	26 (13)
Mitotic inhibitors	9 (14.5)	9 (6.5)	18 (9)
Antimetabolites	32 (51.6)	63 (45.7)	95 (47.5)
Tyrosine kinase inhibitors	3 (4.8)	3 (2.2)	6 (3)
Monoclonal antibody	6 (9.7)	18 (13)	24 (12)
Hormone treatment	0 (0)	1 (0.7)	1 (0.5)
Number of drugs, *n* (%)			
1	18 (38.3)	27 (31.4)	45 (22.5)
2	24 (51.1)	42 (48.8)	66 (33)
≥3	5 (10.6)	17 (19.8)	22 (11)
Central venous catheter, *n* (%)	6 (12.2)	14 (10.9)	20 (11.3)
ECOG performance status, *n* (%)			
0	15 (26.3)	37 (31.9)	52 (30.1)
1	34 (59.6)	65 (56)	99 (57.2)
2	6 (10.5)	10 (8.6)	16 (9.2)
3	1 (1.8)	2 (1.7)	3 (1.7)
4	1 (1.8)	2 (1.7)	3 (1.7)
Performance status ECOG ≤2, *n* (%)	55 (96.5)	112 (96.6)	167 (96.5)
Cancer type, *n* (%)			
Colorectal	21 (33.9)	38 (27.5)	59 (29.5)
Lung	10 (16.1)	25 (18.1)	35 (17.5)
Breast	6 (9.7)	10 (7.2)	16 (8)
Gynecological	5 (8.1)	10 (7.2)	15 (7.5)
Upper gastrointestinal	4 (6.5)	10 (7.2)	14 (7)
Bladder	1 (1.6)	12 (8.7)	13 (6.5)
Hematological	3 (4.8)	4 (2.9)	7 (3.5)
Pancreatic	2 (3.2)	5 (3.6)	7 (3.5)
Kidney	1 (1.6)	4 (2.9)	5 (2.5)
Brain	1 (1.6)	3 (2.2)	4 (2)
Others	8 (12.9)	17 (12.3)	25 (12.5)
Histology, *n* (%)			
Adenocarcinoma	37 (59.7)	73 (52.9)	110 (55)
Epidermoid	3 (4.8)	12 (8.7)	15 (7.5)
Urothelial	1 (1.6)	12 (8.7)	13 (6.5)
Hematological	3 (4.8)	4 (2.9)	7 (3.5)
Oat cell	1 (1.6)	3 (2.2)	4 (2)
Others	17 (27.4)	34 (24.6)	51 (25.5)
Creatinine (mL/min), mean (SD)	0.85 (0.28)	0.87 (0.66)	0.87 (0.57)
Creatinine clearance (mL/min), mean (SD)	83.5 (24.5)	92.9 (65.4)	90 (56.2)
Anticoagulant treatment, *n* (%)			
Enoxaparin	28 (45.2)	64 (46.4)	92 (46)
Tinzaparin	24 (38.7)	46 (33.3)	70 (35)
Bemiparin	9 (14.5)	19 (13.8)	28 (14)
Dalteparin	1 (1.6)	8 (5.8)	9 (4.5)
Vitamin K antagonist	0 (0)	1 (0.7)	1 (0.5)
VTE recurrent, *n* (%)	6 (9.7)	15 (10.9)	21 (10.5)
Bleeding, *n* (%)	5 (8.2)	10 (7.3)	15 (7.5)
Major bleeding, *n* (%)	1 (20)	1 (10)	2 (1)
1-month mortality, *n* (%)	1 (1.6)	2 (1.4)	3 (1.5)
3-month mortality, *n* (%)	2 (3.2)	10 (7.2)	12 (6)
6-month mortality, *n* (%)	9 (14.5)	22 (15.9)	31 (15.5)
Deaths, *n* (%)	28 (45.2)	53 (38.4)	81 (40.5)

Abbreviations: DVT: deep vein thrombosis; SD: standard deviation; VTE: venous thromboembolism; ECOG: Eastern Cooperative Oncology Group.

**Table 2:** Baseline patient characteristics in the no-death vs. death groups.

Variable	No-Deaths (*n* = 119)	With Deaths (*n* = 81)
Male sex, *n* (%)	71 (59.7)	43 (53.1)
Age (years), mean (SD)	65.3 (12.7)	65.4 (12)
Weight (kg), mean (SD)	74.3 (14.5)	72.5 (13.6)
DVT, *n* (%)	34 (28.3)	28 (34.6)
Metastases, *n* (%)	55 (50.5)	60 (78.9)
Oncological Treatment at the Time of a VTE Event, *n* (%)	72 (60.5)	61 (78.2)
Central venous catheter, *n* (%)	15 (13.9)	5 (7.2)
ECOG performance status, *n* (%)		
0	34 (32.7)	18 (26.1)
1	58 (55.8)	41 (59.4)
2	7 (6.7)	9 (13)
3	2 (1.9)	1 (1.4)
4	3 (2.9)	0
ECOG performance status ≤2, *n* (%)	99 (95.2)	58 (98.6)
Cancer type, *n* (%)		
Colorectal	41 (34.5)	18 (22.2)
Lung	17 (14.3)	18 (22.2)
Breast	7 (5.9)	9 (11.1)
Gynecological	9 (7.6)	6 (7.4)
Upper gastrointestinal	10 (8.4)	4 (4.9)
Bladder	7 (5.9)	6 (7.4)
Hematological	5 (4.2)	2 (2.5)
Pancreatic	2 (1.7)	5 (6.2)
Kidney	2 (1.7)	3 (3.7)
Brain	2 (1.7)	2 (2.5)
Others	17 (14.3)	8 (9.9)
Histology, *n* (%)		
Adenocarcinoma	67 (56.3)	43 (53.1)
Epidermoid	8 (6.7)	8 (9.9)
Urothelial	8 (6.7)	6 (7.4)
Hematological	5 (4.2)	2 (2.5)
Oat cell	5 (4.2)	0 (0)
Others	26 (21.9)	22 (27.1)
Creatinine (mL/min), mean (SD)	0.89 (0.7)	0.84 (0.3)
Creatinine clearance (mL/min), mean (SD)	86.4 (24.6)	95.4 (83.1)
Recurrence, *n* (%)	15 (12.6)	6 (7.4)
Bleeding, *n* (%)	6 (5.1)	9 (11.3)
Major bleeding, *n* (%)	1 (16.7)	1 (11.1)

Abbreviations: DVT: deep vein thrombosis; ECOG: Eastern Cooperative Oncology Group SD: standard deviation; VTE: venous thromboembolism.

**Table 3:** Unadjusted and adjusted hazard ratios for overall mortality in patients with incidental pulmonary embolism.

Risk Factor	Unadjusted HR (95% CI)	*p*-Value	Adjusted HR (95% CI)	*p*-Value
Male sex	1.52 (0.6–3.86)	0.383		
Presence of DVT	1.09 (0.43–2.75)	0.855		
Weight, per kilogram	0.95 (0.91–0.99)	0.024	0.96 (0.92–0.99)	0.032
Age, per year	1.03 (0.99–1.01)	0.107		
Central venous catheter	0.28 (0.04–2.09)	0.213		
Creatinine clearance	1 (0.99–1.01)	0.613		
Metastases	9.12 (1.96–42.34)	0.005	10.26 (2.35–44.9)	0.002
Oncological treatment at thrombotic event	2 (0.55–7.3)	0.297		

Abbreviations: CI: confidence interval; DVT: deep vein thrombosis; HR: hazard ratio. Patients evaluated (*n* = 167), deaths (*n* = 23). Analysis was performed using the backward step method (Wald).
